# Renin–angiotensin–aldosterone system activation in plasma as marker for prognosis in critically ill patients with COVID-19: a prospective exploratory study

**DOI:** 10.1186/s13613-025-01433-3

**Published:** 2025-01-16

**Authors:** Katharina Krenn, Felix Kraft, Luana Mandroiu, Verena Tretter, Roman Reindl-Schwaighofer, Theresa Clement, Oliver Domenig, Matthias G. Vossen, Gregor Riemann, Marko Poglitsch, Roman Ullrich

**Affiliations:** 1 Department of Anaesthesia, Intensive Care Medicine and Pain Medicine, Division of General Anaesthesia and Intensive Care Medicine, Medical University of Vienna, Vienna, Austria; 2 Department of Internal Medicine III, Clinical Division of Nephrology and Dialysis, Medical University of Vienna, Vienna, Austria; 3 Division of Nursing Science, Karl-Landsteiner University of Health Sciences, Krems, Austria; 4 Attoquant Diagnostics GmbH, Vienna, Austria; 5 Department of Internal Medicine I, Clinical Division of Infectiology, Medical University of Vienna, Vienna, Austria; 6 Department of Anesthesiology and Intensive Care Medicine, AUVA Trauma Center, Vienna, Austria

**Keywords:** COVID-19, Acute respiratory distress syndrome, Renin–angiotensin system, Aldosterone

## Abstract

**Background:**

Acute respiratory distress syndrome (ARDS) associated with coronavirus infectious disease (COVID)-19 has been a challenge in intensive care medicine for the past three years. Dysregulation of the renin–angiotensin system (RAS) is linked to COVID-19, but also to non-COVID-19 ARDS. It is still unclear whether changes in the RAS are associated with prognosis of severe COVID-19.

**Methods:**

In this prospective exploratory study, blood samples of 94 patients with COVID-19 were taken within 48 h of admission to a medical ward or an ICU. In ICU patients, another blood sample was taken seven days later. Angiotensin (Ang) I-IV, Ang 1–7, Ang 1–5 and aldosterone concentrations were measured with liquid chromatography tandem mass spectrometry (LC–MS/MS) followed by calculation of markers for activities of renin (PRA-S) and ACE (ACE-S), alternative RAS activation (ALT-S) as well as the ratio of aldosterone to Ang II (AA2R). Angiotensin-converting enzyme (ACE) and ACE2 concentrations were measured by LC–MS/MS-based assays. All RAS parameters were evaluated as predictors of 28-day and 60-day survival using receiver operating characteristic and multivariate logistic regression analysis.

**Results:**

AA2R at inclusion was a predictor of 60-day survival for ICU patients with an AUROC of 0.73. Ang II and active ACE2 were inversely associated with survival (OR 0.07; 95%CI 0.01, 0.39 and OR 0.10; 95%CI 0.01, 0.63) while higher Ang 1–7 predicted favorable outcome (OR 6.8; 95%CI 1.5, 39.9). ICU patients showed higher concentrations of all measured angiotensin metabolites, PRA-S, ALT-S and active ACE2, and lower ACE-S and AA2R than patients in the medical ward at inclusion. After seven days in the ICU, Ang I, Ang II, Ang III and Ang IV concentrations decreased, while ACE and ACE2 levels increased. Ang I, PRA-S, Ang 1–7 and Ang 1–5 concentrations correlated with the SOFA score both at the time of inclusion and after seven days, and driving pressure after seven days.

**Conclusions:**

AA2R at inclusion predicted 60-day survival with moderate sensitivity, revealing a dissociation between unchanged aldosterone and increased Ang II levels in the most severely ill COVID-19 patients. After adjustment for confounders, Ang 1–7 as the final metabolite of alternative RAS was predictive for survival.

**Supplementary Information:**

The online version contains supplementary material available at 10.1186/s13613-025-01433-3.

## Background

Acute respiratory distress syndrome (ARDS) may originate from a multitude of direct or indirect injuries to the lung [1, 2]. One cause that has gained worldwide relevance since 2020 is the severe form of the infection with severe acute respiratory syndrome (SARS)-coronavirus (CoV)-2 [3] that may lead to pneumonia and ARDS. Due to the involvement of angiotensin-converting enzyme (ACE) 2, an enzyme of the renin–angiotensin–(aldosterone) system (RAS) as cell surface receptor for SARS-CoV-2 and potential therapeutical target [4–6], the regulation of the RAS in coronavirus infectious disease (COVID)-19 has been subject of study since its emergence.

The RAS is a very complex system of enzymes (peptidases), receptors and biologically active peptides (hormones) regulating mainly volume homeostasis, vascular resistance and blood pressure, but also further processes, such as inflammation, proliferation, angiogenesis and fibrosis [7]. Components of the RAS are found in all major organs despite local differences in abundance and regulation (local tissue RAS, circulatory RAS). RAS hormones form an inter-dependent network of alternatively cleaved peptides, where the equilibrium is determined by enzyme activities and feedback loops, but also crosstalk to other hormone systems and signaling pathways, such as the kallikrein-kinin system, estrogen, cortisol, WNT/ß-catenin signaling and others. Individual peptides seem to have specific biological activities, which are mediated by their genuine target receptors, some of which have only been elucidated recently [8]. The two main branches of the peptide network (classical RAS and alternative RAS) are counter-active, therefore the center of gravity determines outcome. Dysregulation of the RAS is associated with several pathologies encountered in intensive care medicine such as sepsis, vasodilatory shock, acute lung injury, ventilator induced lung injury (VILI) and ARDS [9]. Alterations of enzyme activities and shifts in equilibrium of RAS peptides seem often to be related to disease severity, therefore quantitative assessment of RAS components are considered to have diagnostic and prognostic value.

In this prospective study we used liquid chromatography-tandem mass spectrometry (LC-MSMS) to quantify equilibrium peptide levels (Ang I-IV, Ang 1–7, Ang 1–5) (RAS fingerprint) and active ACE and ACE2 in order to identify their implications for prognosis in COVID-19 patients. This approach allows to further calculate signature ratios (PRA-S, ACE-S, ALT-S, AA2R), correlates of RAS enzyme activities, which otherwise need to be determined by individual enzyme activity tests.

## Patients and methods

### Study design

The study was assessed favorably by the ethics committee of the Medical University of Vienna (protocol number 1328/2020). Patients > 18 years of age, diagnosed with COVID-19 who had been admitted to the medical wards and ICUs dedicated to treatment of patients with COVID-19 at the Medical University of Vienna were included within 48 h of admission. Inclusion criteria were a positive polymerase chain reaction test for SARS-CoV-2 and hospitalization, either in a medical ward or an ICU. During the study period, screening and inclusion of patients was performed in a prospective manner, however, was complicated by shortage of study personnel during the pandemic, especially on weekends, potentially affecting patient selection. Exclusion criteria were cardiogenic pulmonary edema and brain stem death. Patients transferred for evaluation of lung transplantation late in the course of COVID-19 were also excluded. Informed consent was obtained from all study participants according to the Austrian legislation regulating the consent of conscious and nonconscious patients participating in clinical trials. Blood samples were taken within 48 h of admission to the ward or ICU and upon development of ARDS according to the Berlin Definition [1]. In addition, patients transferred to our hospital for evaluation for ECMO therapy were eligible and samples were also obtained within 48 h of transfer. A second blood sample was taken after seven days from ICU patients who met the ARDS definition. Plasma was separated immediately after sampling and stored at -20 °C until analysis.

### Markers of COVID-19 severity in routine laboratory assessment

Albumin, C-reactive protein (CRP), interleukin (IL)-6, ferritin, lymphopenia, lactate dehydrogenase (LDH) and D-dimer have been described in correlation with severity of disease [2, 10] as well as development of refractory pneumonia [11] and ARDS [12]. Routinely assessed albumin, lymphocyte count and percentage, platelet count, LDH, D-dimer, CRP, procalcitonin and interleukin (IL)-6 levels were documented on the day of study sampling. Ferritin levels were available only for patients admitted to the ICU.

### Quantification of angiotensin metabolites

Angiotensin metabolite equilibrium concentrations in heparin plasma were measured by liquid chromatography tandem mass spectrometry (LC–MS/MS) with the capability to analyze angiotensin peptide concentrations with a lower limit of quantification (LLOQ) of 2 to 4 pmol/L for individual metabolites (Attoquant Diagnostics GmbH, Vienna, Austria). In this study, equilibrium concentrations of Ang I, Ang II, Ang III, Ang IV, Ang 1–7, and Ang 1–5 as well as aldosterone levels (LLOQ 10 pmol/L) were investigated.

The plasma was conditioned for equilibrium analysis at 37 °C, followed by stabilization as described previously [13]. The equilibrated samples were then spiked with stable isotope-labeled internal standards for each angiotensin metabolite at a concentration of 200 pg/mL. The samples subsequently underwent C-18 based solid-phase-extraction and were subjected to LC–MS/MS analysis, using a reversed-phase analytical column operating in line with a Xevo TQ-S triple quadruple mass spectrometer (Waters, Milford, MA, USA). Internal standards were used to correct for peptide and steroid recovery of the sample preparation procedure for each analyte in each individual sample. Analyte concentrations were reported in pmol/L.

### Calculation of angiotensin-based markers of RAS enzyme activities

Markers for renin and ACE activity as well as alternative RAS activation were PRA-S (Ang I + Ang II concentrations), which is highly correlated with plasma renin activity [14, 15], ACE-S (Ang II/Ang I ratio) for ACE activity [14] and ALT-S [(Ang 1–7 + Ang 1–5)/(Ang I + Ang II + Ang 1–7 + Ang 1–5)] [16] for activation of the alternative RAS axis. The AA2R was calculated as the aldosterone/Ang II concentration ratio [15].

### Active ACE and ACE2 concentrations

The active ACE concentration was determined in diluted plasma samples (phosphate-buffered saline, pH 7.4) after spiking samples with Ang I and ex vivo incubation at 37 °C in the presence (inhibitor) and absence (solvent) of the ACE inhibitor lisinopril (10 µmol/L, Sigma-Aldrich, Munich, Germany). Aminopeptidase inhibitor (10 µmol/L, Sigma-Aldrich), Z-Pro-prolinal (10 µmol/L, Sigma-Aldrich) and ACE2 inhibitor MLN-4760 (10 µmol/L, Sigma-Aldrich) were added to all samples for substrate (Ang I) and product (Ang II) stabilization. The quantification of Ang I and Ang II was performed as described above by LC–MS/MS. The specific activity of ACE was calculated by determining the inhibitor sensitive fraction (solvent minus inhibitor) of Ang II formation. To calculate the active ACE concentration, the specific ACE activity in the sample was related to the activity of recombinant human ACE (R&D Systems, Minneapolis, USA).

The active ACE2 concentration was measured as described [17]. Plasma samples were diluted and pre-conditioned with an Ang 1–7 stabilizing protease inhibitor mix. After spiking with Ang II, samples were incubated at 37 °C for 60 min followed by quantification of Ang II and Ang 1–7. The Ang 1–7 formation rate was calculated in [(pmol/L)/h], which was subsequently converted to ng/mL, using the linear calibration obtained from a standard curve of ACE2 in human healthy volunteer plasma. The Ang II levels were compared to non-incubated control samples to verify that the substrate was present in excess during the incubation period, ensuring a stable Ang 1–7 formation rate during the incubation period.

### Determination of active renin by ELISA

PRA-S is considered a correlate ratio indicating the activity of the primary RAS enzyme renin, which has been shown in the context of other disease encountered in ICUs to be a predictor of mortality [18, 19]. We therefore analyzed active renin concentration by ELISA in a subgroup of 41 samples from our ICU patient cohort in order to verify statistical correlation between ELISA read-outs and PRA-S calculated from our RAS fingerprint analysis. Active renin was determined from plasma samples by a sandwich ELISA (DRG Instruments GmbH) with a dynamic range of 0.8–128 pg/ml according to instructions of the manufacturer and finally correlated with PRA-S.

### Multiplex ELISA (Luminex)

Plasma levels of the following analytes were determined with a Magnetic Luminex Assay (R&D Systems; cat.nr. LXSAHM): Angiopoietin-2 (Angpt2), interferon (INF)-γ, interleukin-8 (IL-8), receptor for advanced glycation end products (RAGE), surfactant protein-D (SP-D), and TNF receptor-I (TNFRI). Plasma samples previously stored at − 20 °C were thawed, centrifuged for 10 min at 16,000*g*, diluted twofold in the calibrator diluent RD6-52 provided with the kit, and processed according to the manufacturer’s instructions. The custom-defined standard cocktail was reconstituted and a series of threefold dilutions was prepared for the standard curve. The diluted standards and samples were incubated with the microparticle cocktail for 2 h at room temperature on a horizontal orbital microplate shaker set at 800 rpm. Using a magnetic device, the plates were washed three times with wash buffer and incubated with biotin antibody for one hour at room temperature on the shaker. After 3 washes the beads were incubated with streptavidin-PE for 30 min at room temperature on the shaker. After 3 final washes, the beads were resuspended in 100 µL wash buffer and read on a Luminex MAGPIX analyzer to establish the median fluorescence intensity. A standard curve was prepared using a suitable five-parameter logistic (5-PL) curve to determine analyte concentrations.

### Statistical analysis

IBM SPSS statistics 28.0.1.0, GraphPad Prism 9.1.1 and R 4.3.1 were used for data analysis. The primary outcome, the ability of RAS parameters to predict survival on day 28 and 60 in ICU patients, was assessed by univariate binary logistic regression analysis and receiver operating characteristic (ROC) analysis. After assessment for collinearity, selected RAS parameters were then analyzed as independent variables in multivariate logistic regression models adjusted for potential confounders including age, gender, SOFA, ECMO therapy at inclusion and history of arterial hypertension with stepwise backward elimination for survival at day 28 and day 60. Due to the unpredictable sample size of the study during the pandemic, the selection of confounders was limited to the most important ones in the perception of the authors. The survival of ICU patients until day 60 after inclusion was plotted as Kaplan–Meier curves stratified by AA2R below and above the best cut-off from ROC analysis. Secondary outcomes, i.e. differences between the patient groups from the medical ward and the ICU at inclusion and ICU patients at inclusion and seven days later were analyzed with the Mann Whiney-U test and Wilcoxon signed rank test, respectively. Correlations between clinical parameters such as SOFA score, driving pressure and ARDS severity and RAS parameters were analyzed with Spearman’s rank test. The correlations, logistic regression and ROC analyses were performed using log_10_ transformed RAS data to eliminate the effect of non-normal distributions. If a concentration was below the LLOQ, the respective LLOQ divided by square root of two was used [20, 21]. A subgroup analysis of RAS metabolites and clinical patient characteristics was performed for patients with ECMO therapy for ARDS at inclusion, as these patients represented more than half of the ICU cohort. Statistical analyses were performed as exploratory analyses without adjustment for multiple testing and *P*-values < 0.05 were considered significant.

## Results

### Study population

Twenty patients admitted to the medical ward and 75 patients admitted to the ICU were included into the study. One initially nonconscious ICU patient declined participation later and was excluded. The patients were included during the first wave as well as the alpha, delta and omicron variant waves in Austria between May 2020 and February 2022. Patient characteristics and comorbidities are summarized in Table 1.

**Table 1:** Demographics and comorbidities of hospitalized patients with COVID-19 at the medical ward or the ICU

Variable	Medical ward (*N* = 20)	ICU (*N* = 74)
Age, years	61 ± 20	53 ± 10
Gender (m/f), n (%)	12/8 (60/40)	52/22 (70/30)
BMI, kg/m^2^	28.0 ± 6.6^a^	32.7 ± 6.4
WHO score *Respiratory support, n (%)*	4.0 ± 0.8	6.9 ± 0.3
IMV NIV + HFNO O_2_ insufflation room air	0 5 (25) 8 (40) 7 (35)	73 (99) 1 (1) 0 0
Acute kidney injury, n (%)^b^	4 (20.0)	19 (26)
Time of inclusion, days after hospital admission ICU admission intubation	2 (1–3)^c,d^	9 (5–15) 6 (2–11) 3 (2–8)^c,d^
*Medical history, n (%)*		
Arterial hypertension	12 (60)	43 (58)
Diabetes mellitus type II	6 (30)	19 (26)
BMI > 30 kg/m^2^	5 (36)^a^	46 (62)
Heart disease^e^	8 (40)	12 (16)
Lung disease^e^	1 (5)	10 (14)
Chronic kidney disease	6 (30)	6 (8)
Neurologic disease^e^	6 (30)	5 (7)
Depression	1 (5)	11 (15)
*Home medication, n (%)*		
None	2 (10)	19 (26)
ACEi	0	10 (14)
ARB	5 (25)	14 (19)
Betablocker	3 (15)	17 (23)
Calcium channel blocker	4 (20)	8 (11)
Diuretic	2 (10)	12 (16)
Statin	5 (25)	10 (14)
Immunosuppression	2 (10)	8 (11)
Psychotropic drugs	1 (5)	15 (20)
Data on home medication missing	4 (20)	13 (18)

Data are expressed as number and percentages, mean and standard deviation or median and interquartile range, depending on the scale and distribution of the variable.

*BMI* body mass index, *WHO score* World health organization ordinal scale, *IMV *invasive mechanical ventilation, *NIV* non-invasive ventilation, *HFNO* High-flow nasal oxygen therapy, *ACEi* Angiotensin converting enzyme inhibitor, *ARB* Angiotensin receptor blocker

^a^BMI was not available in six patients

^b^Defined as any stage of acute kidney injury according to the KDIGO classification

^c^Two patients included after transfer to a COVID-19 medical ward due to diagnosis of COVID-19 from within hospital

^d^Time spans include admission and intubation at an external ICU. ^e^Detailed information on diagnoses of heart, lung, and neurologic diseases are listed in supplemental Table S7

Patients admitted to the medical ward had a mean WHO score of 4.0 at inclusion and none were treated in an ICU later. One patient died at the medical ward without transfer to an ICU because of expected futility (geriatric patient with immobility due to neurological condition). All other patients were discharged from the hospital and survived beyond day 60. All ICU patients met ARDS criteria upon admission to the ICU and had a mean WHO score of 6.9 at inclusion. As the study was conducted at a referral center for ECMO therapy and lung transplantation, forty-three (58%) of the ICU patients were transferred from other hospitals for evaluation for ECMO therapy. This treatment had already been started in 33 of these 43 patients at inclusion in the study < 48 h after transfer. Seven of these referred patients were stabilized with inhaled nitric oxide (NO), and did not immediately require ECMO therapy, while three patients stabilized without ECMO or inhaled NO. Seven days later, ECMO therapy had been initiated in three more patients, while three patients had died despite ECMO therapy.

Table 2 shows the clinical condition, ventilation parameters and supportive therapies of ICU patients at inclusion and seven days later.

**Table 2:** Clinical parameters in ICU patients with COVID-19 at inclusion and after seven days

Variable	ICU at inclusion (*N* = 74)	ICU after 7 days (*N* = 62^a^)
SOFA score	10.8 ± 2.3	9.8 ± 3.3
*Catecholamines and inotropics*		
Norepinephrine dose^b^, max mcg/kg/min in 24 h	0.041 (0.015–0.086)	0.022 (0.000–0.047)
Norepinephrine, n (%)	59 (80)	36 (58)
Inotropics, n (%)	17 (23)	1 (2)
*ARDS Severity by Berlin definition (1), n (%)*		
No ARDS	0	4 (6)
Mild	10 (14)	9 (15)
Moderate	35 (47)	33 (53)
Severe	29 (39)	16 (26)
*Ventilation mode, n (%)*		
Pressure controlled	54 (73)	20 (31)
Airway pressure release vent	1 (1)	2 (3)
Partially assisted	19 (26)	29 (47)
Pressure support	0	10 (16)
NIV	1 (1)	0
Nasal cannula	0	2 (3)
*Respiratory parameters*		
Peak ventilation pressure, mbar	28 ± 4	24 ± 6
Driving pressure, mbar	15 ± 4	13 ± 4
PEEP, mbar	13 ± 2	12 ± 3
PaO_2_/FiO_2_, mmHg	135 (103–170)	159 (120–199)
PaCO_2_, mmHg	47.5 ± 10.7	45.4 ± 7.9
Respiratory rate, min^−1^	17 ± 5	19 ± 7
VT, mL/kg PBW	5.5 ± 2.2	5.2 ± 2.5
Compliance,^c^ mL/mbar	27.1 ± 11.3	24.1 ± 15.3
*Supportive therapies, n (%)*		
Renal replacement therapy	5 (7)	7 (11)
ECMO	38 (51)	38 (61)
NO inhalation	55 (74)	44 (71)
Corticosteroid therapy	64 (87)	41 (66)

Data are expressed as numbers and percentages, mean and standard deviation or median and interquartile range according to the distribution and scale of the variable

*SOFA* sequential organ failure assessment score, *PEEP* positive end-expiratory pressure, *VT* tidal volume, *PBW* predicted body weight, *ECMO* extracorporeal membrane oxygenation, *NO* nitric oxide

^a^Six patients died within 7 days, 3 were transferred to another hospital, and in 3 patients a follow-up sample was not available

^b^Noraldrenalin dose is reported as base formulation equivalent

^c^Only calculated in patients on controlled ventilation

A total of 38 of 74 patients (51%) received ECMO therapy, of whom 37 patients had a venovenous ECMO cannulation for acute respiratory failure, while one case with concomitant myocarditis received peripheral venoarterial ECMO. Fifty-five patients (74%) received inhaled NO and five patients (7%) required renal replacement therapy at inclusion. ECMO and inhaled NO were applied at the same time in 32 of these patients (43%). Nineteen patients (26%) developed acute kidney injury during the ICU stay. Patients admitted to the ICU had significantly higher levels of CRP, IL-6, procalcitonin, d-dimer, LDH, and white blood cell counts than patients in the medical ward, while albumin levels and percentage of lymphocytes in differential blood count were lower (Table S1).

After 28 days, patients treated at the medical ward had a mean WHO score of 1.5 ± 1.7, and after 60 days the WHO score was 1.4 ± 1.6. In contrast, patients admitted to an ICU had a WHO score of 6.4 ± 1.8 after 28 days and a WHO score of 5.8 ± 2.6 after 60 days. One (5%) of 20 patients in the medical ward and 17 (23%) of 74 patients in the ICU died within 28 days and an additional 9 ICU patients died within 60 days of inclusion without leaving the ICU (60-day mortality 35%). Patients requiring ECMO therapy at inclusion had a 28- and 60-day mortality of 29% and 42%, while patients without ECMO at inclusion had a 28- and 60-day mortality of 17% and 28% (Table S2). Four patients requiring prolonged ECMO therapy due to non-recovering severe structural lung disease following COVID-19 pneumonia underwent a lung transplantation. Sixteen patients were still in the ICU on day 60 after inclusion.

### Pattern of systemic RAS activation in patients with COVID-19 ARDS

**Fig. 1:**
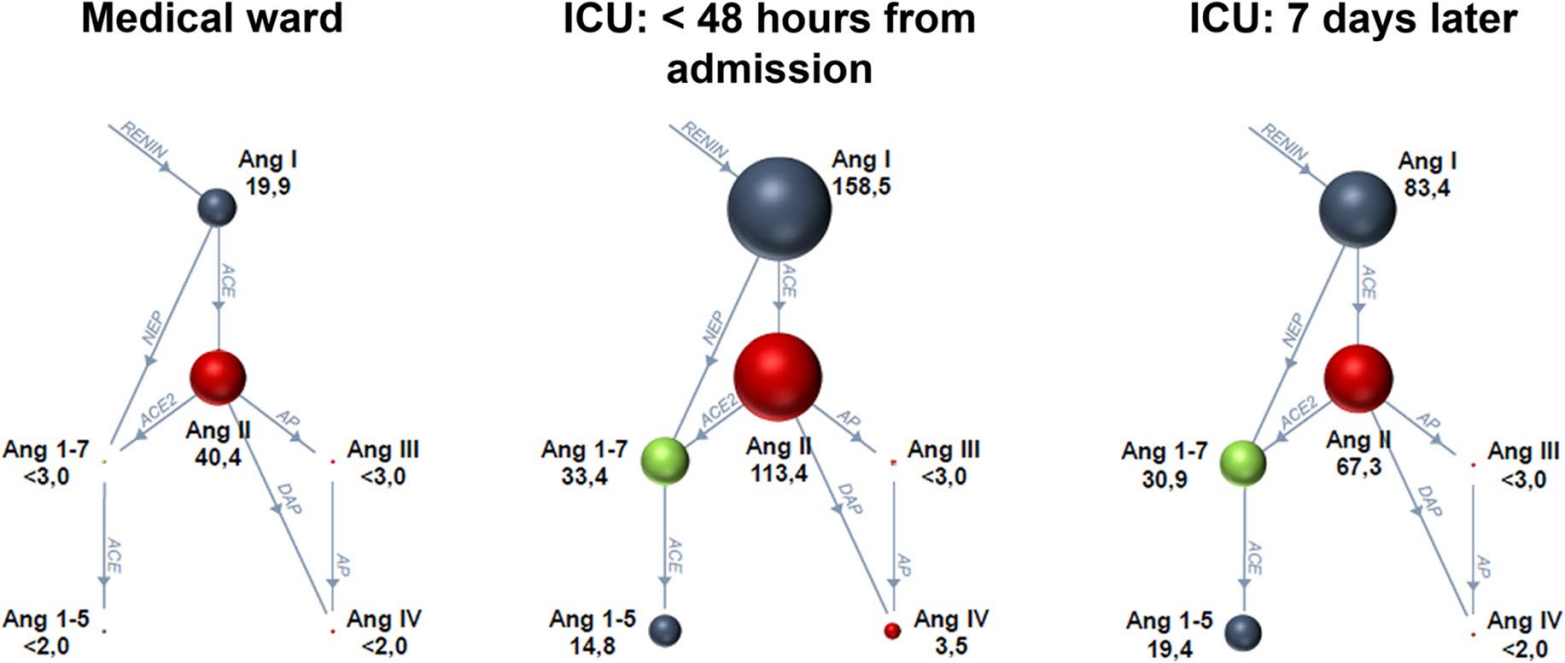
Renin–angiotensin system fingerprints of patients with COVID-19 treated in the medical ward at inclusion and patients with COVID-19 treated in the ICU at inclusion and after seven days. Data are shown as medians and sphere sizes correspond to the respective median angiotensin metabolite concentrations. *Ang* angiotensin, *ACE* angiotensin-converting enzyme, *AP* aminopeptidase, *NEP* neprilysin, *DAP* diaminopeptidase

All measured angiotensin metabolite concentrations (Ang I-IV, Ang 1–7 and Ang 1–5) as well as active ACE2 levels in plasma were elevated in patients admitted to the ICU versus patients in the medical ward (all *P* < 0.05, Table 3). Angiotensin metabolite concentrations (Ang I-IV, Ang 1–7 and Ang 1–5) are shown as RAS-fingerprints in Fig. 1.

**Table 3:** Renin–angiotensin system parameters in plasma in patients with COVID-19 admitted to the medical ward or the ICU

Variable	Medical ward (*N* = 20)	ICU at inclusion (*N* = 74)	ICU after 7 days (*N* = 62^a^)
*RAS component*			
Ang I, pmol/L	19.9 (12.1–89.9)	158.5 (52.9–552.0)**	83.4 (28.0–214.4)^§§^
Ang II, pmol/L	40.4 (14.6–118.6)	113.4 (41.6–388.3)*	67.3 (28.5–164.5)^§^
Ang 1–7, pmol/L	< 3 (< 3–8.6)	33.4 (4.6–98.8)***	30.9 (6.9–61.5)
Ang 1–5, pmol/L	< 2 (< 2–7.2)	14.8 (4.7–56.8)***	19.4 (5.3–44.3)
Active ACE, µg/mL	6.7 (4.7–7.8)	6.4 (5.0–8.2)	7.7 (5.6–9.3)^§§§^
Active ACE2, ng/mL	1.7 (1.4–2.4)	8.3 (4.3–18.2)***	12.9 (7.0–32.5)^§§§^
Ang III, pmol/L	< 3 (< 3- < 3)	< 3 (< 3–8.5)**	< 3 (< 3- < 3)^§^
Ang IV, pmol/L	< 2 (< 2–3.8)	3.5 (< 2–10.3)*	< 2 (< 2–4.1)^§§^
Aldosterone, pmol/L	51.7 (19.7–95.3)	44.9 (15.3–119.0)	54.9 (13.3–114.7)
Renin, pg/mL		28.0 (10.9–54.8)^†^	
*Markers and ratios*			
PRA-S, pmol/L	60.9 (26.0–324.9)	315.2 (93.7–1126.2)**	167.5 (65.4–443.6)^§§^
ACE-S	2.19 (1.02–3.10)	0.95 (0.54–1.58)**	1.12 (0.64–1.65)
ALT-S	0.06 (0.04–0.11)	0.13 (0.09–0.24)**	0.23 (0.14–0.35)^§§§^
AA2R	1.66 (0.85–3.04)	0.40 (0.17–1.05)***	0.65 (0.23–1.55)^§^

Data are expressed as median and interquartile range

*RAS* Renin–angiotensin system, *Ang* angiotensin, *ACE* angiotensin-converting enzyme, *PRA-S* (Ang I + Ang II) concentrations as marker of renin activity, *ACE-S* Ang II/Ang I ratio, ALT-S: [(Ang 1–7 + Ang 1–5)/(Ang I + Ang II + Ang 1–7 + Ang 1–5)] as marker of alternative renin–angiotensin system activation, AA2R: Aldosterone/Ang II ratio

^a^Six patients died within 7 days, 3 were transferred to another hospital, and in 3 patients a follow-up sample was not available. *P < 0.05, **P < 0.01, ***P < 0.001 versus medical ward, by Mann–Whitney U-test, ^§^*P* < 0.05, ^§§^*P* < 0.01, ^§§§^*P* < 0.001 versus ICU at inclusion, by Wilcoxon signed rank test. ALT-S could be calculated in *N* = 10 (50%) in the medical ward, and *N* = 64 (88%) and *N* = 57 (90%) in the ICU at inclusion and after 7 days, respectively. ^†^Renin concentration was available in *N* = 37 (50%) patients in the ICU at inclusion

Renin activity represented by PRA-S and alternative RAS activation indicated by ALT-S were higher (*P* = 0.005 and *P* = 0.008, respectively), while ACE activity, indicated by ACE-S, was decreased in patients admitted to the ICU versus patients in the medical ward (*P* = 0.002, Table 3). In addition, the AA2R was decreased in patients in the ICU compared to patients in the medical ward (*P* < 0.001). This difference in the AA2R was due to higher Ang II levels in ICU patients that were accompanied by unchanged aldosterone levels compared to patients in the medical ward.

In ICU patients, a second sample was taken after seven days, in which Ang I (*P* = 0.008), Ang II (*P* = 0.011), PRA-S (*P* = 0.005), Ang III (*P* = 0.022) and Ang IV levels (*P* = 0.007) had decreased compared to the first measurement (Table 3), which indicates declining classical RAS activation. However, the levels of the alternative RAS metabolites Ang 1–7 and Ang 1–5 remained unchanged, and ACE and ACE2 levels as well as ALT-S were increasing (all *P* < 0.001 versus day of inclusion). The AA2R also increased after seven days compared to the day of inclusion (*P* = 0.028), but remained much lower in patients in the ICU than in the medical ward. It is also of note that median Ang I levels were still higher than median Ang II levels in ICU patients after seven days, to the effect that ACE-S stayed below the values observed in patients in the medical ward.

### Evaluation of RAS parameters for prediction of 28-day and 60-day survival

The primary endpoint of this exploratory study was to evaluate the RAS parameters at inclusion as predictive markers of prognosis for 28-day and 60-day survival. As all patients already presented with ARDS at the time of ICU admission, and participating patients in the medical ward did not require ICU admission, the analyses were only performed on the group of patients with COVID-19 ARDS admitted to an ICU (*N* = 74). Although no RAS parameter predicted 28-day survival in univariate binary logistic regression analysis, Ang II, PRA-S, Ang IV, Ang 1–5, and AA2R at inclusion predicted 60-day survival (all *P* < 0.05, Table S3). Also, no demographic or clinical parameter predicted 28-day survival. Age and SOFA score at inclusion predicted 60-day survival (Table S3). Active renin concentration measured in a subset of ICU patients (n = 37 within range of standard curve) was closely correlated to PRA-S (r = 0.805, *P* < 0.001; Supplemental Figure S1) without predictive value in univariate logistic regression for both 28-day and 60-day survival (Table S3).

In a multivariate binary logistic regression model including potential confounders (age, gender, arterial hypertension, ECMO therapy, SOFA) with Ang II, Ang 1–7, ACE2, and AA2R, only age, Ang II, Ang 1–7, and ACE2 remained significant predictors of 60-day survival (Table 4). Especially Ang 1–7 was positively associated with 60-day survival with an OR of 6.8 (95% CI 1.49, 39.92; p = 0.021), while higher Ang II levels were negatively associated with 60-day survival (OR 0.07; Table 4). No association was found with 28-day survival for the same initial model selection.

**Table 4:** Multivariate logistic regression analysis for 60-day survival on RAS parameters within 48 h of ICU admission

Variable	Selected model	
	*OR (95% CI)*	*P*
Age	0.92 (0.85, 0.98)	**0.016**
SOFA score	0.672 (0.43, 0.97)	0.051
Ang II	0.070 (0.01, 0.39)	**0.006**
Ang 1–7	6.774 (1.49, 39.92)	**0.021**
ACE2	0.098 (0.01, 0.63)	**0.025**

*Ang* angiotensin, *ACE* angiotensin-converting enzyme, *ECMO* extracorporeal membrane oxygenation, *SOFA* sequential organ failure assessment score, *AA2R* aldosterone/Ang II ratio

Bold values for *P* indicate statistical significane with p<0.05 in multivariate logistic regression

The full model consisted of RAS parameters selected after testing for collinearity, as well as confounders potentially affecting the RAS as well as outcome in ICU patients (Table S4). After backward elimination, the selected model remained

**Fig. 2:**
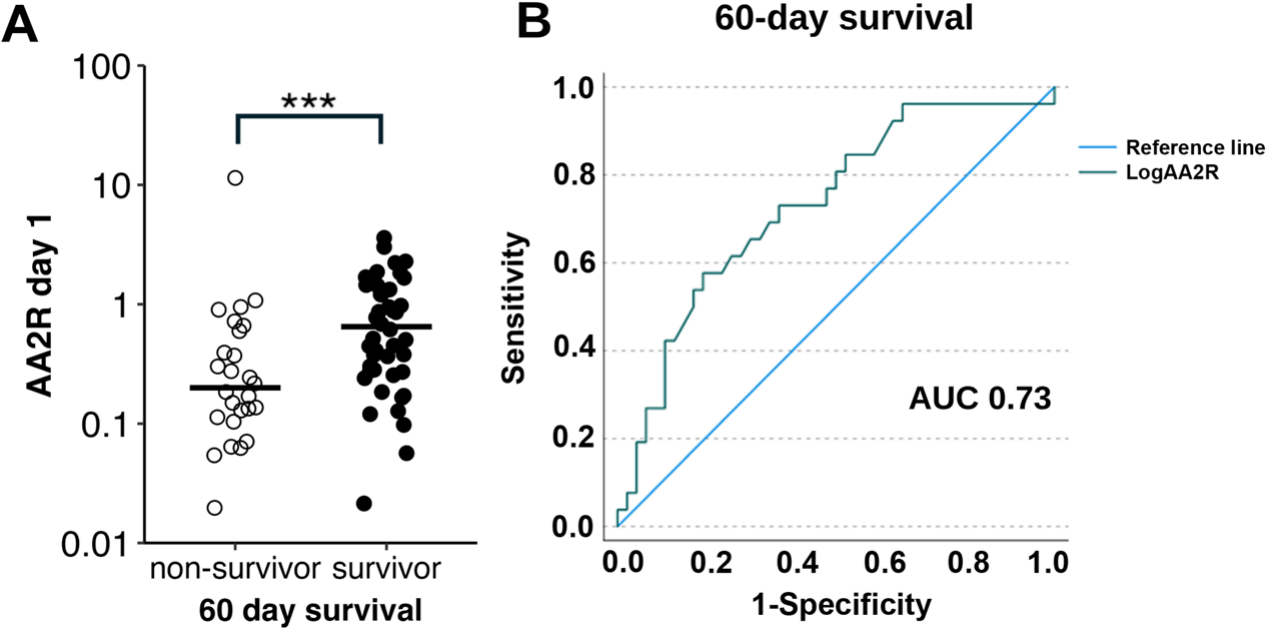
Relationship between aldosterone/angiotensin II ratio (AA2R) and 60-day survival in ICU patients with COVID-19. **A** Patients who did not survive until day 60 (non-survivors) had lower AA2R values than patients surviving until day 60 (survivors), ***P = 0.001, compared by Mann–Whitney-U test. **B** Receiver operating characteristic curve of AA2R at a cut-off ≥ 0.40 for 60-day survival with an area under the curve (AUC) of 0.73

Non-survivors had lower initial AA2R values than survivors (0.20 vs. 0.65; *P* = 0.001, Fig. 2A). At a cut-off ≥ 0.40, the AA2R at inclusion had a sensitivity of 73.1% and specificity of 63.0% to predict 60-day survival with an AUC of 0.73 in ROC analysis (Fig. 2B), which outperformed the SOFA score at a cut-off < 11 at inclusion (sensitivity 73.1%, specificity 41.7%, AUC = 0.65 for 60-day survival).

**Fig. 3:**
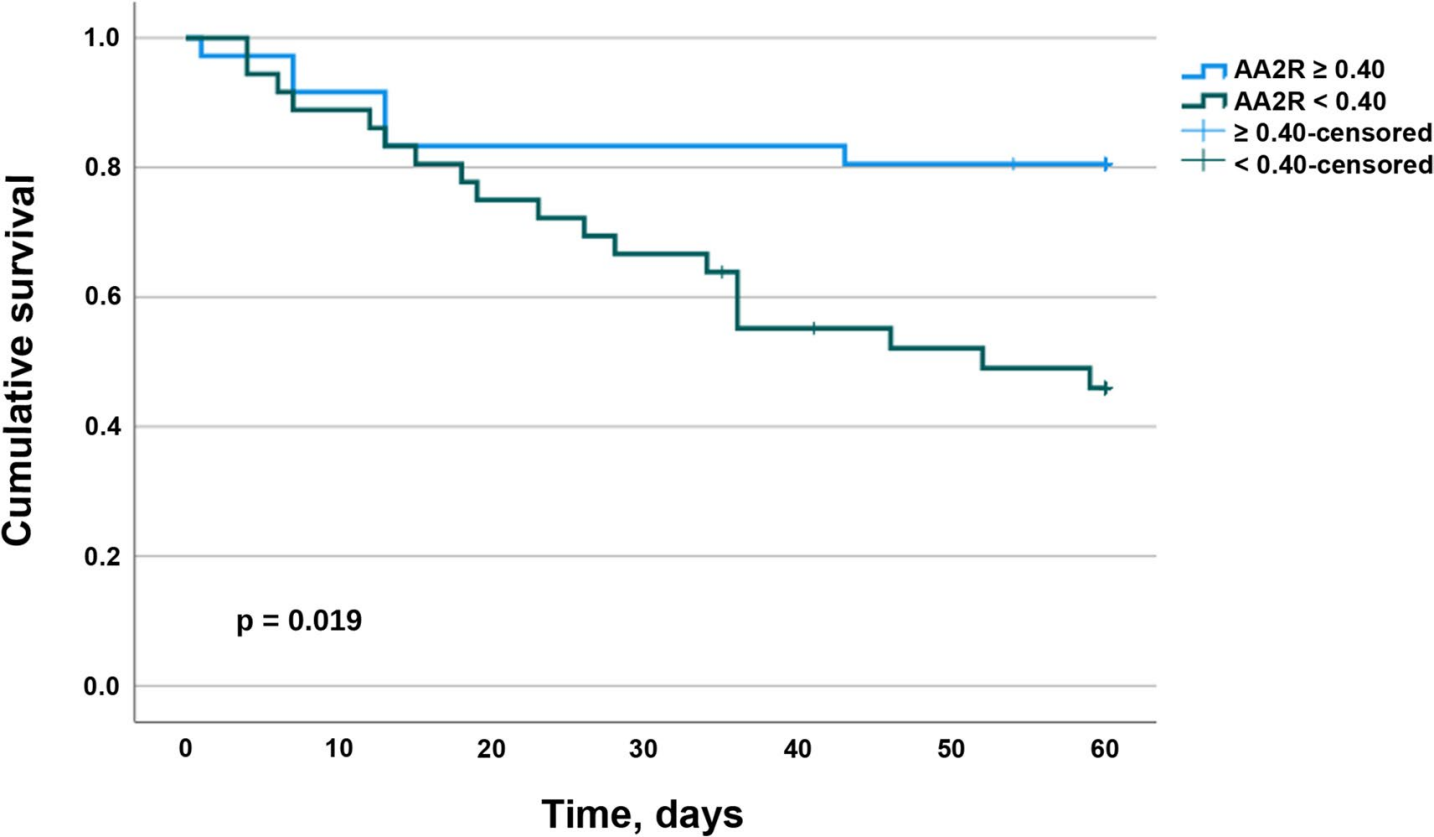
Kaplan–Meier curve of ICU patients with COVID-19 for 60 days stratified by AA2R at inclusion. Patients with an aldosterone to angiotensin II ratio (AA2R) of 0.4 or greater had a significantly higher survival probability 60 days after intensive care unit admission (p = 0.019; log-rank test)

The difference in survival according to AA2R < or ≥ 0.40 at inclusion was also evident in a stratified Kaplan–Meier plot (Fig. 3).

### Correlation of RAS parameters with clinical condition in ICU patients

**Fig. 4:**
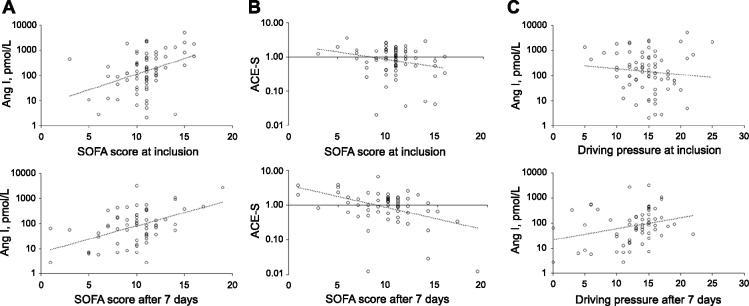
Exemplary correlations of renin–angiotensin system parameters with SOFA score and driving pressure. Correlation of Angiotensin I (**A**) and ACE-S (Ang II/Ang I ratio, **B**) with SOFA score, and Ang I with driving pressure (**C**) at inclusion and after seven days

On the day of inclusion Ang I, Ang II, PRA-S, Ang IV, Ang 1–7, and Ang 1–5 concentrations directly correlated with the SOFA score (r = 0.256–0.384, all *P* < 0.05). Seven days later, Ang I, PRA-S, Ang 1–7, and Ang 1–5 still directly correlated with the SOFA score on that day (r = 0.275–0.392, all *P* < 0.05). In addition, ACE-S (r = -0.341, *P* = 0.007) and AA2R (r = -0.412, *P* < 0.001) showed an indirect correlation with the SOFA score after seven days, but not initially. Exemplary correlations between SOFA score and Ang I and ACE-S at inclusion and seven days later are shown in Fig. 4A, B, respectively. ARDS severity at inclusion, expressed as PaO_2_/FiO_2_ ratio, was negatively correlated with active ACE2 (r = -0.294, *P* = 0.013).

At the time of inclusion, no ventilation parameter correlated with any RAS parameter in ICU patients. However, Ang I, PRA-S, Ang 1–7, and Ang 1–5 concentrations directly correlated with the driving pressure (r = 0.275–0.372, all *P* < 0.05) and peak inspiratory pressure (r = 0.286–0.409, all *P* < 0.05) seven days later. As an example, the correlation between Ang I concentrations and driving pressure is shown in Fig. 4C. There was also an indirect correlation between ACE-S and the peak inspiratory pressure (r = -0.316, *P* = 0.013) after seven days, but not initially.

The maximum norepinephrine dose required on the day of inclusion directly correlated with Ang I, Ang II, PRA-S, Ang III, Ang IV, Ang 1–7, Ang 1–5, and aldosterone concentrations (r = 0.241–0.338, all *P* < 0.05). Seven days later, only aldosterone levels and the AA2R correlated indirectly with the maximum norepinephrine dose on that day (r = -0.258, *P* = 0.043, and r = -0.410, *P* < 0.001, respectively).

At inclusion, patients receiving ECMO therapy had lower AA2R values (0.28 [IQR 0.13–0.79]) than patients without ECMO (0.61 [IQR 0.28–1.45], *P* = 0.017). The other RAS parameters did not differ between ICU patients with and without ECMO therapy at inclusion (Table S5).

### Plasma levels of ARDS biomarkers

At inclusion, IL-8, Angpt2 and SP-D levels were higher in ICU patients than in patients in the medical ward (Table S6, all *P* < 0.01). Plasma levels of RAGE were not different between ICU patients and patients in the medical ward at inclusion and decreased 7 days after inclusion in ICU patients (*P* = 0.019 versus day of inclusion). Plasma levels of TNFRI were higher in patients with an AA2R ≥ 0.40 (*P* = 0.006), while the other ARDS biomarkers were not different between patients with an AA2R above or < 0.40.

## Discussion

Previous studies have shown, that activation of the RAS is a frequently observed issue in ICU patients. Systemic RAS activation occurs in non-COVID-19 ARDS [22–24] and ventilator-induced lung injury (VILI) [16, 25], and may also be reflected in the RAS activation pattern in mechanically ventilated patients with COVID-19 ARDS. Therefore, several studies have attempted to relate RAS key enzyme activities and metabolite levels to clinical parameters and outcome in order to offer measures, that might have predictive potential early in treatment.

Angiotensin (Ang) I levels and ACE activity (derived from the Ang II/Ang I concentration ratio) in plasma may serve as prognostic markers in early ARDS [22, 23], and Ang I levels have been shown to correlate with driving pressure after one week of ARDS [23]. ACE activity is reduced in patients with COVID-19 ARDS compared to non-COVID-19 ARDS especially in finally non-surviving patients [26, 27], and in patients with severe versus non-severe COVID-19 [28]. Soluble ACE2 levels have been found elevated or increasing over time in patients with more severe COVID-19 [28, 29], while other studies could not confirm this finding [30, 31]. Contradictory results were also found for aldosterone in COVID-19. Some studies reported no change in aldosterone levels in SARS-CoV-2 positive compared to negative patients at emergency departments [31, 32], other studies found lower [33] or higher [34] aldosterone levels in patients with more severe COVID-19.

Explanations for these discrepancies might be differences in disease severity and analytical methodology [35]. Many of these studies used immunoassays, which may be subject to interference in patients with COVID-19 [22], and the timing of the first measurement varied from first presentation at the emergency department [31, 32] or “early after admission” [32] to within 48 h of hospital or ICU admission [33], highlighting the importance of meticulous description of methodology, study timeline and patient characteristics.

In our prospective study we included a group of almost exclusively mechanically ventilated ICU patients with COVID-19, including a high percentage (51%) of patients with ECMO therapy at inclusion, and a group of patients with COVID-19 treated in the medical ward.

Among all RAS parameters measured at inclusion, the aldosterone/Ang II ratio, i.e. the AA2R, turned out to be the most accurate predictor of 60-day survival in ICU patients with a moderate sensitivity and specificity, while no RAS parameter predicted 28-day survival. After adjustment for selective confounders in the multivariate model, Ang 1–7 appeared highly predictive for survival, while AA2R was not significantly associated with outcome anymore. The association of increased Ang 1–7 levels with outcome was assessed in recent clinical trials of intravenous administration of Ang 1–7 in COVID-19 ICU patients. Ang 1–7 showed no consistent benefits in regard to liberation from respiratory support or mortality [36, 37]. Still, these studies reported no major side effects while doses and severity of disease between studies were markedly different, which justifies further investigation of a potential therapeutic use of Ang 1–7.

In the patients requiring ICU treatment, we observed RAS activation patterns which are indicative of ARDS and COVID-19 [22, 23, 28].

The angiotensin metabolite profiles of the ICU patients with COVID-19 ARDS in this study showed a larger increase in Ang I than in Ang II levels leading to reduced ACE-S (Ang II/Ang I ratio) that were also observed in non-COVID-19 ARDS [22, 23] and in patients with severe COVID-19 [28] in earlier studies.

Concentrations of Ang I, Ang 1–7, and Ang 1–5 correlated with ventilation pressures seven days after inclusion, which has also been observed in patients with non-COVID-19 ARDS [23]. This may indicate that prolonged mechanical ventilation with higher pressures and persisting severity of disease is also associated with RAS activation in COVID-19 ARDS. RAS findings typical of severe COVID-19 included increased levels of the alternative RAS metabolites Ang 1–7 and Ang 1–5 [28] together with increased soluble ACE2 levels [29, 38] and ALT-S [28]. While increased ACE2 levels have also been observed in non-COVID-19 ARDS [23, 24], their association with disease severity [29, 38, 39] and their impact on circulating alternative RAS metabolite levels and ALT-S [28] seem to be more pronounced in COVID-19 ARDS. This association was also present in our study, with higher active ACE2 levels in more severe ARDS accompanied with lower PaO_2_/FiO_2_ ratios and a negative prediction for survival of active ACE2 in the multivariate analysis. El-Shennawy et al. have shown that extracellular vesicles expressing ACE2 are increased in plasma of patients with COVID-19, and may be related to disease severity [40]. If ACE2 was located on the surface of these vesicles and was enzymatically active, it would have also been detected by the active ACE2 assay used in our study.

Despite critical illness with respiratory and hemodynamic compromise, aldosterone levels unexpectedly remained unchanged in ICU patients with COVID-19 ARDS versus patients in the medical ward and were unproportionally low compared to their elevated Ang II levels, which resulted in a reduced AA2R. Most interestingly, the AA2R turned out to be the most accurate predictor of 60-day survival of patients with COVID-19 ARDS in the ICU, driven by these increased Ang II levels as shown as well in the multivariate analysis. In patients receiving ECMO, the median AA2R at inclusion was below the cut-off found for 60-day survival and was the sole parameter that differed significantly compared to ICU patients without ECMO, based on a combination of lower aldosterone levels with higher Ang II levels. However, the AA2R did not correlate with the SOFA score at inclusion. Earlier studies have described the aldosterone/renin ratio in COVID-19 patients [33, 34, 41], but our study was the first to describe the AA2R in patients with COVID-19. Measured renin concentrations in a subset of patients admitted to the ICU were not associated with outcome, which contrasts with data on critically ill patients with and without COVID-19 [42]. This might be in part explained by the relatively low renin levels in the present study, as reported thresholds for significant associations with mortality lay two- to three fold higher. Additionally, the elevated Ang II levels might have inhibited in part renin release in this cohort.

The ICU cohort reported here is one of the largest to be investigated for RAS activation in this amount of detail to date and is unique because 58% of patients were referred for ECMO evaluation shortly after initial external ICU admission, and 99% were on invasive mechanical ventilation. Earlier studies only involved limited numbers of patients with severe COVID-19 or ICU admission. In one study only 6 of 15 patients with severe COVID-19 received invasive mechanical ventilation [34], while the other studies either provided no information on mechanical ventilation of ICU patients [33] or no information on the ventilation mode (invasive versus noninvasive) [41]. It is therefore unclear how these data compare to our results of 74 critically ill patients with COVID-19, 99% of whom were invasively mechanically ventilated at inclusion. Another study measured aldosterone levels with LC–MS/MS and found levels below the LLOQ (70 pmol/L) in more than half of the patients [35]. Since only 12 of 134 patients needed invasive mechanical ventilation in this study, no comparison of aldosterone levels with and without mechanical ventilation was feasible. As the RAS is activated in VILI [16, 25] and in ARDS [22, 23], this may have influenced renin and aldosterone levels in patients with COVID-19 in these studies and, apart from methodological differences [35], may explain the differing results that aldosterone levels were lower [33] or higher [34] in patients with severe COVID-19. In our study the LLOQ for aldosterone measured by LC–MS/MS was 10 pmol/L, yet the aldosterone levels at inclusion were below this LLOQ in 3/20 patients in the medical ward and 16/74 patients in the ICU. One factor contributing to the relatively low aldosterone levels in ICU patients may have been ongoing corticosteroid therapy in the majority (87%) of our patients at inclusion. While this may explain the similarity of aldosterone levels in ICU patients and patients in the medical ward (who also received corticosteroids in 70%), the AA2R was even lower in ICU patients who did not survive until day 60. Another explanation for the low aldosterone levels in relation to Ang II concentrations in ICU patients may be negative regulation of aldosterone secretion by the increased Ang 1–7 levels [43].

The direct correlations between angiotensin metabolite concentrations and PRA-S with the SOFA score and maximum norepinephrine dose on the day of inclusion and the SOFA score after seven days in these most severely ill COVID-19 patients indicate their critical condition, when renin may play a role as an indicator of tissue hypoperfusion [44].

In contrast to our study, the groups defined as having severe or critical COVID-19 in other studies on the RAS in COVID-19 included proportionally more patients on NIV or high flow nasal oxygen cannula (HFNO) [26–28, 32, 34, 35, 45, 46], or the authors did not specify the ventilation mode [30, 31, 34, 47, 48]. As VILI [16, 25] and PEEP [49] are drivers of RAS activation, the description of the ventilation mode (invasive mechanical ventilation versus NIV/HFNO) is of importance in studies on RAS activation involving patients with all types of ARDS due to its potential impact on the angiotensin metabolite profile. In our study, the five patients receiving HFNO or NIV in the medical ward showed heterogeneous findings, but their median Ang II levels were higher than Ang I levels (as opposed to those in ICU patients), and all presented with an AA2R > 0.40.

As a limitation, the protocol of this study was written in March 2020, when not much was known about the course of severe COVID-19. At the time, there were no plans for further sampling in addition to seven days after inclusion in ICU patients. Only few patients were admitted to an ICU from the emergency department and most patients (58%) were transferred to our center for evaluation for ECMO therapy due to the central ICU bed allocation in Vienna during the study period. This is reflected in the length of time between ICU admission and inclusion in ICU patients, and “inclusion < 48 h after ICU admission” was defined as < 48 h after admission at our center. By chance, none of the participants in the medical ward needed ICU treatment later, which meant that no sequential sampling was performed in deteriorating patients. The number of patients recruited in the medical ward was too small for testing outcomes. Furthermore, there has been a selection bias favoring the age group < 65–70 years in the group of ICU patients, because 58% of ICU patients were transferred from other hospitals to be evaluated for ECMO therapy, and thus needed to be eligible for this treatment according to national guidelines. The majority of patients (87%) were already receiving a corticosteroid when admitted to the ICU, which meant that the effect of this therapy on aldosterone levels could not be further clarified. Due to their critical condition, none of the ICU patients with regularly prescribed ACE inhibitors or angiotensin receptor blockers had taken a RAS blocker at least within the last 48 h before ICU admission at our center. Only two patients were treated with an ACE inhibitor or angiotensin receptor blocker seven days after inclusion, meaning that the effects of these therapies on the results were expected to be marginal.

The assessed covariates in the multivariate logistic model were chosen to represent frequently used confounders of ICU morbidity and mortality (age and SOFA score), as well as RAS components representing both, the alternative and the classical axis, after testing for collinearity. However, with a final model of five independent variables and only limited events and sample size, overfitting of the model might be an issue. Also, certain factors, especially related to ECMO application, were not taken into account in the model, but might have influenced outcome significantly. These limitations need to be carefully considered on interpretation.

## Conclusions

Among the RAS parameters investigated in this study, an initial AA2R ≥ 0.40 turned out to be the most accurate predictor of 60-day survival in ICU patients with COVID-19 ARDS, while the reasons for the unproportionally low aldosterone levels in non-survivors of ICU treatment as well as patients receiving ECMO remain unclear.

Several features of the angiotensin metabolite profile in ICU patients with COVID-19 ARDS overlap with those found in mechanically ventilated patients with non-COVID-19 ARDS, such as a larger increase in Ang I than in Ang II levels leading to reduced ACE-S, and correlation of ventilation parameters with angiotensin levels after seven days. This study further confirmed that increased Ang 1–7, Ang 1–5, and ALT-S are frequently observed in COVID-19 ARDS, indicating an increased activation of the alternative RAS axis. Correlations between the SOFA score, maximum norepinephrine dose, ventilation pressures and angiotensin metabolite levels underscore the fact that high RAS activation is an indicator of more severe critical illness. The protective effects of increased Ang 1–7 shown after adjusting for selective confounders have to be carefully assessed in future trials, as its role as a biomarker or therapeutic target is not clearly understood yet.

## Supplementary Information

Supplementary fiile 1.

## Data Availability

The datasets analyzed during the current study are available from the corresponding author on reasonable request.

## Abbreviations

AA2R: Aldosterone/Angiotensin II ratio
ACE: Angiotensin-converting enzyme
ACE-S: Angiotensin II/Angiotensin ratio as marker of angiotensin-converting enzyme activity
ALT-S: Angiotensin metabolite concentration-based marker for alternative RAS activation
Ang: Angiotensin
Angpt: Angiopoietin
ARDS: Acute respiratory distress syndrome
COVID-19: Coronavirus infection disease of 2019
ECMO: Extracorporeal membrane oxygenation
HFNO: High-flow nasal oxygen
IL: Interleukin
INF: Interferon
LLOQ: Lower limit of quantification
LC–MS/MS: Liquid chromatography tandem mass spectrometry
NIV: Noninvasive ventilation
PRA-S: Angiotensin I + Angiotensin II concentrations, marker of renin activity
RAGE: Receptor for advanced glycation end products
RAS: Renin–angiotensin system
ROC: Receiver operating curve
SARS-CoV-2: Severe acute respiratory syndrome coronavirus 2
SOFA: Sequential organ failure assessment score
SP-D: Surfactant protein D
TNFRI: Tumor necrosis factor receptor I
WHO: World health organization
