# Supplementary material for: Renin–angiotensin–aldosterone system activation in plasma as marker for prognosis in critically ill patients with COVID-19: a prospective exploratory study
Source: Ann Intensive Care. 2025 Jan 16;15:10. doi: 10.1186/s13613-025-01433-3 (PMC11739446; doi:10.1186/s13613-025-01433-3)
Supplement: Supplementary file 1 — Supplementary fiile 1. [file 13613_2025_1433_MOESM1_ESM.docx]

**Supplemental material**

**Renin-angiotensin-aldosterone system activation in plasma as marker for prognosis in critically ill patients with COVID-19: A prospective exploratory study**

Katharina Krenn, MD, PhD^1*^; Felix Kraft, MD^1^; Luana Mandroiu, MD^1^; Verena Tretter, PhD^1^; Roman Reindl-Schwaighofer, MD, PhD^2^; Theresa Clement, MSc^1,3^; Oliver Domenig, PhD^4^; Matthias G. Vossen, MD, PhD^5^; Gregor Riemann, BSc^1^; Marko Poglitsch, PhD^4^; Roman Ullrich, MD^1,6^

^1^Department of Anesthesia, General Intensive Care and Pain Medicine, Medical University of Vienna, Vienna, Austria

^2^Department of Internal Medicine III, Clinical Division of Nephrology and Dialysis, Medical University of Vienna, Vienna, Austria

^3^Division of Nursing Science, Karl-Landsteiner University of Health Sciences, Krems, Austria

^4^Attoquant Diagnostics GmbH, Vienna, Austria

^5^Department of Internal Medicine I, Clinical Division of Infectiology, Medical University of Vienna, Vienna, Austria

^6^Department of Anesthesiology and Intensive Care Medicine, AUVA Trauma Center, Vienna, Austria

# Figures

**Figure S1.** Correlation of serum renin concentration with PRA-S (renin activity) at inclusion in patients admitted to an intensive care unit with COVID-19 ARDS.

Pearson correlation of renin concentration in serum and renin activity (PRA-S), calculated as Ang I+Ang II, of ICU patients at inclusion. PRA-S is highly correlated with serum renin. PRA-S was measured with Liquid chromatography tandem mass spectrometry (LC-MS/MS) and renin was measured using enzyme-linked immunosorbent assay (ELISA). This analysis was performed in a subset of 37 randomly chosen ICU patients from the study collective.

# Tables

**Table S1.** Laboratory indicators of severity of COVID-19 in hospitalized patients at the medical ward and the ICU

| **Laboratory parameters** | **Medical ward**  (*N* = 20) | **ICU at inclusion**  (*N* = 74) | **ICU after 7 days**  (*N* = 62^a^) |
| --- | --- | --- | --- |
| CRP, mg/dL | 2.48 (0.65-10.88) | 14.25 (9.95-22.84) | 7.46 (3.84-13.92) |
| IL-6, pg/mL | 22.0 (10.9-29.3) | 66.0 (31.5-147.0)*** | 45.8 (32.3-196.0) |
| Procalcitonin | 0.09 (0.07-0.13) | 0.65 (0.22-1.90)*** | 0.23 (0.16-0.51)§ |
| Albumin | 36.6 (29.1-41.4) | 26.1 (22.9-28.7)*** | 27.3 (24.6-30.5) |
| WBC count, G/L | 6.10 (4.55-7.26) | 11.12 (7.98-14.30)*** | 8.30 (6.56-12.53)§§ |
| Lymphocytes, G/L | 1.0 (0.6-1.4) | 0.7 (0.5-1.0) | 1.1 (0.8-1.7)§§§ |
| Lymphocytes, % | 16 (12-27) | 7 (4-12)*** | 13 (8-19)§§§ |
| Platelet count, G/L | 196 (146-287) | 238 (166-322) | 199 (137-286)§§ |
| D-dimer | 0.7 (0.4-0.8) | 3.6 (2.2-8.3)*** | 3.8 (1.9-9.5) |
| LDH, U/L | 285 (241-398) | 423 (345-570)*** | 373 (312-487)§§ |
| Bilirubin, mg/dL | 0.36 (0.26-0.67) | 0.60 (0.40-1.00) | 0.55 (0.40-1.00) |
| Creatinine, mg/dL | 0.92 (0.79-1.35) | 0.78 (0.64-1.18) | 0.69 (0.53-0.99)§ |
| Ferritin | N.A. | 1123.8 (605.0-1846.7) | 970.4 (481.2-1834.5) |

Data are expressed as median and interquartile range. ***P<0.001 versus medical ward, by Mann-Whitney-U test. §p < 0.05, §§p < 0.01, §§§p < 0.001 versus ICU at inclusion, by Wilcoxon signed rank test. N.a.: not available. aSix patients died within 7 days, 3 were transferred to another hospital, and in 3 patients a follow-up sample was not available. CRP: C-reactive protein, IL: interleukin, WBC: white blood cell, LDH: lactate dehydrogenase.

**Table S2.** Characteristics and outcomes in patients with COVID-19 stratified by ECMO therapy

| **Variable** | **no ECMO at ICU inclusion** (*N* = 36) | **ECMO therapy at ICU inclusion** (*N* = 38) | *P* |
| --- | --- | --- | --- |
| *Demographics* |  |  |  |
| Age, years | 51 (45, 61) | 56 (50, 61) | 0.203 |
| Gender (m/f), n (%) | 9/27 (25/75) | 13/25 (34/66) | 0.386 |
| BMI, kg/m^2^ | 30.9 (27.2, 38.3) | 31.3 (29.6, 36.0) | 0.991 |
| WHO Score | 6.8 ± 0.5 | 7.0 ± 0 | 0.009 |
| SOFA Score | 10.6 | 11.0 | 0.938 |
| PaO_2_/FiO_2_, mmHg | 139 (107, 157) | 133 (102, 184) | 0.957 |
| ARDS classification pre ECMO, n (%) |  |  |  |
| mild | N/A | 1 (2.6) |  |
| moderate | N/A | 4 (11) |  |
| severe | N/A | 33 (87) |  |
|  |  |  |  |
| ARDS classification at inclusion, n (%) |  |  | 0.002 |
| mild | 5 (14) | 5 (13) |  |
| moderate | 24 (67) | 11 (29) |  |
| severe | 7 (19) | 22 (58) |  |
|  |  |  |  |
| Respiratory support n, (%) |  |  | 1 |
| IMV | 35 (97.2) | 36 (100) |  |
| NIV | 1 (2.8) | 0 (0) |  |
|  |  |  |  |
| *Medical history, n (%)* |  |  |  |
| Arterial hypertension | 219 (53) | 24 (63) | 0.366 |
| Diabetes mellitus type II | 6 (17) | 13 (34) | 0.084 |
| Obesity (BMI >30gk/m^2^) | 21 (58) | 25 (66) | 0.509 |
| Heart disease | 5 (14) | 7 (18) | 0.597 |
| Lung disease | 5 (14) | 5 (13) | 1 |
| Chronic kidney disease | 3 (8.3) | 3 (7.9) | 1 |
| Neurologic disease | 2 (5.6) | 3 (7.9) | 1 |
| Depression | 1 (2.8) | 10 (26) | 0.004 |
| Malign disease | 2 (5.6) | (0) | 0.233 |
| Hematologic disease | 0 (0) | 2 (5.3) | 0.494 |
| History of transplantation | 1 (2.8) | 3 (7.9) | 0.615 |
| *Home medication, n (%)* |  |  |  |
| None |  |  |  |
| ACEi | 3 (8.3) | 7 (18) | 0.310 |
| ARB | 5 (14) | 9 (24) | 0.282 |
| Beta blocker | 8 (22) | 9 (24) | 0.881 |
| Calcium channel blocker | 3 (8.3) | 5 (13) | 0.712 |
| Diuretics | 5 (14) | 7 (18) | 0.597 |
| Statins | 4 (11) | 6 (16) | 0.737 |
| Immunosuppression | 4 (11) | 4 (11) | 1 |
| Psychotropic drugs | 3 (8.3) | 11 (29) | 0.024 |
| Data on home medication missing | 6 (17) | 12 (32) | 0.135 |
|  |  |  |  |
| *Outcomes* |  |  |  |
| Acute kidney injury, n (%)^b^ | 10 (28) | 9 (24) | 0.687 |
| Survival at day 28 | 30 (83) | 27 (71) | 0.209 |
| Survival at day 60 | 26 (72) | 22 (58) | 0.197 |
| ICU survival | 22 (61) | 21 (55) | 0.610 |

^b^Defined as any stage of acute kidney injury according to the KDIGO classification. BMI: body mass index, WHO score: World health organization ordinal scale, SOFA score: sequential organ failure assessment score, ARDS: acute respiratory distress syndrome, IMV: invasive mechanical ventilation, NIV: non-invasive ventilation, ACEi: Angiotensin converting enzyme inhibitor, ARB: Angiotensin receptor blocker, ICU: intensive care unit.

**Table S3.** RAS and demographic and clinical parameters at inclusion (within 48 hours of ICU admission) in univariate logistic regression analysis to predict outcome

| **Variable** | **28-day survival** |  | **60-day survival** |  |
| --- | --- | --- | --- | --- |
|  | *OR (95% CI)* | *P* | *OR (95% CI)* | *P* |
| *RAS component* |  |  |  |  |
| Ang I, pmol/L | 0.732 (0.34, 1.48) | 0.395 | 0.508 (0.25, 0.97) | 0.051 |
| Ang II, pmol/L | 0.707 (0.32, 1.53) | 0.382 | 0.431 (0.19, 0.88) | **0.027*** |
| Ang 1-7, pmol/L | 0.934 (0.46, 1.90) | 0.849 | 0.609 (0.31, 1.15) | 0.133 |
| Ang 1-5, pmol/L | 0.770 (0.35, 1.68) | 0.511 | 0.449 (0.21, 0.92) | **0.034*** |
| Active ACE, µg/mL | 0.667 (0.02, 13.32) | 0.804 | 0.900 (0.05, 13.67) | 0.940 |
| Active ACE2, ng/mL | 0.884 (0.25, 3.06) | 0.845 | 0.705 (0.23, 2.10) | 0.531 |
| Ang III, pmol/L | 0.716 (0.27, 2.02) | 0.504 | 0.496 (0.19, 1.22) | 0.128 |
| Ang IV, pmol/L | 0.614 (0.26, 1.49) | 0.269 | 0.421 (0.18, 0.94) | **0.040*** |
| Aldosterone, pmol/L | 1.321 (0.54, 3.39) | 0.550 | 0.923 (0.41, 2.06) | 0.843 |
| Renin, pg/mL^§^ | 1.000 (0.990, 1.020) | 0.815 | 1.000 (0.980, 1.010) | 0.525 |
| *Markers and ratios* |  |  |  |  |
| PRA-S, pmol/L | 0.695 (0.31, 1.48) | 0.355 | 0.457 (0.21, 0.92) | **0.037*** |
| ACE-S | 0.953 (0.24, 3.15) | 0.941 | 0.862 (0.26, 2.58) | 0.795 |
| ALT-S | 3.401 (0.64, 20.41) | 0.157 | 2.095 (0.46, 10.35) | 0.342 |
| AA2R | 2.828 (0.99, 8.98) | 0.061 | 4.351 (1.6, 13.68) | **0.007*** |
| *Demographic and clinical parameters* |  |  |  |  |
| Age | 0.948 (0.89, 1.00) | 0.086 | 0.923 (0.87, 0.97) | **0.008*** |
| Gender | 0.667 (0.17, 2.19) | 0.526 | 0.811 (0.27, 2.29) | 0.698 |
| History of arterial hypertension | 0.962 (0.31, 2.87) | 0.946 | 0.626 (0.23, 1.66) | 0.352 |
| SOFA | 0.789 (0.59, 1.02) | 0.083 | 0.701 (0.52, 0.90) | **0.011*** |
| ECMO | 0.491 (0.15, 1.47) | 0.214 | 0.529 (0.20, 1.38) | 0.199 |

In this exploratory analysis, *P*-values < 0.05 were considered significant*. ^§^Renin concentration was available in *N* = 37 (50%) patients in the ICU at inclusion. Ang: Angiotensin, ACE: angiotensin-converting enzyme, PRA-S: (Ang I+Ang II), ACE-S: (Ang II/Ang I), ALT-S: [(Ang 1-7+Ang 1-5)/(Ang I+Ang II+Ang 1-7+Ang 1-5)], ACE: Angiotensin converting enzyme, ECMO: extracorporeal membrane oxygenation, SOFA: sequential organ failure assessment score, AA2R: Aldosterone/Ang II ratio.

**Table S4.** Multivariate logistic regression analysis for 60-day survival on RAS parameters within 48 hours of ICU admission before backward elimination

| **Variable** | **Full model** |  |
| --- | --- | --- |
|  | *OR (95% CI)* | *P* |
|  |  |  |
| Age | 0.929 (0.86, 1.00) | 0.054 |
| Gender | 0.948 (0.21, 4.13) | 0.943 |
| History of arterial hypertension | 0.673 (0.19, 2.27) | 0.526 |
| ECMO therapy | 0.824 (0.24, 2.79) | 0.754 |
| SOFA score | 0.668 (0.42, 0.97) | 0.054 |
| Ang II | 0.086 (0.01, 0.059 | **0.020** |
| Ang 1-7 | 6.535 (1.35, 39.95) | **0.027** |
| ACE2 | 0.10 (0.01, 0.67) | **0.029** |
| AA2R | 1.66 (0.41, 7.39) | 0.487 |

The full model consisted of RAS parameters selected after testing for collinearity, as well as confounders potentially affecting the RAS as well as outcome in ICU patients. Ang: Angiotensin, ACE: angiotensin-converting enzyme, ECMO: extracorporeal membrane oxygenation, SOFA: sequential organ failure assessment score, AA2R: Aldosterone/Ang II ratio.

**Table S5**. Renin-angiotensin system parameters in plasma in ICU patients with COVID-19 ARDS stratified by ECMO therapy at inclusion.

| **Variable** | **no ECMO at ICU inclusion** (*N* = 36) | **ECMO therapy at ICU inclusion** (*N* = 38) | *P* |
| --- | --- | --- | --- |
| *RAS component* |  |  |  |
| Ang I, pmol/L | 157.2 (34.0, 552.0) | 158.5 (73.0, 462.5) | 0.7 |
| Ang II, pmol/L | 86.2 (22.5, 260.9) | 125.8 (59.7, 416.8) | 0.2 |
| Ang 1-7, pmol/L | 31.4 (2.7, 98.8) | 37.6 (7.4, 87.2) | 0.8 |
| Ang 1-5, pmol/L | 14.2 (3.5, 50.4) | 17.2 (6.6, 56.8) | 0.6 |
| Active ACE, µg/mL | 6.1 (5.0, 8.0) | 6.6 (5.4, 8.2) | 0.8 |
| Active ACE2, ng/mL | 8.4 (4.8, 18.0) | 8.3 (4.0, 16.4) | >0.9 |
| Ang III, pmol/L | 2.1 (2.1, 6.4) | 2.1 (2.1, 11.2) | 0.7 |
| Ang IV, pmol/L | 2.3 (1.4, 6.2) | 4.1 (1.7, 15.0) | 0.2 |
| Aldosterone, pmol/L | 72.0 (17.5, 136.0) | 35.3 (14.0, 96.4) | 0.3 |
| Renin, pg/mL^§^ | 30.0 (11.5, 69.6); *N=14* | 27.3 (11.0, 54.8); *N=23* | >0.9 |
| *Markers and ratios* |  |  |  |
| **PRA-S**, pmol/L | 287.0 (85.5, 985.43) | 354.9 (134.8, 1,254.9) | 0.5 |
| **ACE-S** | 0.99 (0.52, 1.58) | 0.95 (0.58, 1.48) | >0.9 |
| **ALT-S** | 0.16 (0.09, 0.26) | 0.12 (0.09, 0.19) | 0.4 |
| **AA2R** | 0.61 (0.29, 1.45) | 0.28 (0.13, 0.75) | **0.015** |

Data are expressed as median and interquartile range. **P*<0.05 by Mann-Whitney-U test.

^§^Renin concentration was available in *N* = 37 (50%) patients in the ICU at inclusion. Ang: Angiotensin, ACE: angiotensin-converting enzyme, PRA-S: (Ang I+Ang II), ACE-S: (Ang II/Ang I), ALT-S: [(Ang 1-7+Ang 1-5)/(Ang I+Ang II+Ang 1-7+Ang 1-5)], ACE: Angiotensin converting enzyme, ECMO: extracorporeal membrane oxygenation, SOFA: sequential organ failure assessment score, AA2R: Aldosterone/Ang II ratio.

**Table S6.** Biomarkers of ARDS and inflammation in hospitalized patients with COVID-19 at the medical ward and the ICU

| *ARDS biomarkers* | **Medical ward** (*N* = 18) | **ICU at inclusion** (*N* = 48) | **ICU after 7 days** (*N* = 35) |
| --- | --- | --- | --- |
| IL-8, pg/mL | 16.6 (11.0-21.7) | 40.4 (25.2-81.6)*** | 42.7 (24.8-114.7) |
| Angpt2, pg/mL | 1757 (1239-3209) | 3625 (2153-5277)** | 3936 (2182-5671) |
| IFNγ, pg/mL | 12.1 (8.1-16.0) | 15.9 (12.5-21.0) | 15.0 (13.9-19.6) |
| SP-D, pg/mL | 6204 (3513-25510) | 30311 (19614-41240)*** | 25467 (18217-38049) |
| TNFRI, pg/mL | 2277 (1770-3966) | 3369 (2268-4389) | 3433 (2728-4719) |
| RAGE, pg/mL | 2788 (1580-7112) | 1743 (1134-8111) | 1192 (635-2213)§ |

Data are expressed as median and interquartile range, statistical analysis was performed with Wilcoxon signed-rank test. ***P* < 0.01, ****P* < 0.001 versus medical ward, ^§^*P* < 0.05 versus at inclusion. Number of available samples for biomarker measurement per group is indicated as *N*. IL: interleukin, Angpt: angiopoietin, IFN: interferon, SP: surfactant protein, TNFRI: tumor necrosis factor receptor I, RAGE: receptor of advanced glycation end products.

**Table S7.** Detailed comorbidities summarized as heart, lung and neurologic diseases of included patients

| **Diseases and diagnoses** | **Frequency of diagnoses (N)** |
| --- | --- |
| *Heart disease* |  |
| Coronary heart disease | 9 |
| Atrial fibrillation or other arrhythmia | 11 |
| Pacemaker or implantable cardioverter-defibrillator | 5 |
| Congestive heart failure | 2 |
| History of myocardial infarction | 1 |
|  |  |
| *Lung diseases* |  |
| Asthmatic disease | 6 |
| Sarcoidosis | 2 |
| Chronic inflammatory condition or COPD | 3 |
| Lung fibrosis | 1 |
|  |  |
| *Neurologic disease* |  |
| Cerebrovascular disease | 6 |
| Polyneuropathy | 2 |
| Epilepsy | 2 |
| Parkinson’s disease | 1 |

COPD: chronic obstructive pulmonary disease
